# Landscape of genomic diversity and host adaptation in *Fusarium graminearum*

**DOI:** 10.1186/s12864-017-3524-x

**Published:** 2017-02-23

**Authors:** Benoit Laurent, Magalie Moinard, Cathy Spataro, Nadia Ponts, Christian Barreau, Marie Foulongne-Oriol

**Affiliations:** INRA, UR1264 Mycologie et Sécurité des Aliments, bâtiment Qualis, 71 avenue Edouard Bourlaux, CS 20032, F-33882 Villenave d’Ornon cedex, France

**Keywords:** Fungal pathogen, Fusarium head blight, Whole genome re-sequencing, Genome-wide polymorphism, Single nucleotides polymorphism, Host-Pathogen interaction, Evolution, Two-speed genome

## Abstract

**Background:**

*Fusarium graminearum* is one of the main causal agents of the Fusarium Head Blight, a worldwide disease affecting cereal cultures, whose presence can lead to contaminated grains with chemically stable and harmful mycotoxins. Resistant cultivars and fungicides are frequently used to control this pathogen, and several observations suggest an adaptation of *F. graminearum* that raises concerns regarding the future of current plant disease management strategies. To understand the genetic basis as well as the extent of its adaptive potential, we investigated the landscape of genomic diversity among six French isolates of *F. graminearum*, at single-nucleotide resolution using whole-genome re-sequencing.

**Results:**

A total of 242,756 high-confidence genetic variants were detected when compared to the reference genome, among which 96% are single nucleotides polymorphisms. One third of these variants were observed in all isolates. Seventy-seven percent of the total polymorphism is located in 32% of the total length of the genome, comprising telomeric/subtelomeric regions as well as discrete interstitial sections, delineating clear variant enriched genomic regions- 7.5 times in average. About 80% of all the *F. graminearum* protein-coding genes were found polymorphic. Biological functions are not equally affected: genes potentially involved in host adaptation are preferentially located within polymorphic islands and show greater diversification rate than genes fulfilling basal functions. We further identified 29 putative effector genes enriched with non-synonymous effect mutation.

**Conclusions:**

Our results highlight a remarkable level of polymorphism in the genome of *F. graminearum* distributed in a specific pattern. Indeed, the landscape of genomic diversity follows a bi-partite organization of the genome according to polymorphism and biological functions. We measured, for the first time, the level of sequence diversity for the entire gene repertoire of *F. graminearum* and revealed that the majority are polymorphic. Those assumed to play a role in host-pathogen interaction are discussed, in the light of the subsequent consequences for host adaptation. The annotated genetic variants discovered for this major pathogen are valuable resources for further genetic and genomic studies.

**Electronic supplementary material:**

The online version of this article (doi:10.1186/s12864-017-3524-x) contains supplementary material, which is available to authorized users.

## Background

The ascomycete *Fusarium graminearum (*teleomorphe *Gibberella zeae*) is a hemibiotrophic pathogen commonly described as one of the main causal agent of the Fusarium Head blight (FHB), a devastating disease affecting small grains cereals worldwide [1]. In addition to the defect on annual yield, major concerns arise from contamination of grains by stable and harmful fungal metabolites so-called mycotoxins which are present in feed and food constitute a real threat for consumers and livestock [2]. Molecules belonging to the type B family of trichothecenes (TCTB) are probably the most concerning due to their frequent occurrence and demonstrated toxic effects [3]. The genes acting in TCTB production, named *Tri* genes, are clustered for the majority and expressed after plant penetration with an implication in pathogenicity [4, 5]. Despite the wide array of trichothecenes potentially produced by *F. graminearum isolates*, the spectrum of production observed in individual strains is more limited, defining chemotypes [6, 7]. To date, three chemotypes of TCTB-producing isolates have been described according to their ability to produce deoxynivalenol along with 15-acetyldeoxynivalenol (DON/15-ADON), deoxynivalenol and 3-acetyldeoxynivalenol (DON/3-ADON), and nivalenol and acetylated form (NIV). These chemotypes are associated with quantitative difference in pathogenicity; the strains producing DON instead of NIV are, for example, more aggressive against wheat [8]. In some cases, levels of TCTB have also been found to be correlated with the visual symptoms on the spike [9, 10]. Nevertheless, other factors were identified in *F. graminearum* with the detection of 50 quantitative trait nucleotides linked to aggressiveness variation [11].

Cultivars resistant against FHB and mycotoxin accumulation as well as fungicides are frequently used to control this pathogen [12]. However, there is now evidence that *F. graminearum* is adapting to such strategies, as demonstrated by the emergence of fungicide-resistant strains [13, 14] and the rapid shift towards more aggressive isolates in some part of the world [15]. Cultural management practices must therefore keep up with the “arm race”, which requires a detailed knowledge of the fungus adaptive potential with a special focus on the evolution of pathogenicity-related traits.

Grounds for *F. graminearum* adaptation are certainly provided for by intensive gene flow and large amounts of genetic diversity between and within field populations [16–24]. In *F. graminearum* specifically, these elements are further supported by particular biological features that favor the emergence of genetic diversity, namely a mixed reproduction system based on clonality, selfing and outcrossing [16, 24, 25] as well as both local and long range dispersal of the different spores produced [26–30]. Such combination is indeed particularly efficient to create new haplotypes of which the favorable ones will rapidly spread [31]. The molecular mechanisms underlying the emergence of more aggressive isolates of *F. graminearum* remains remain sparsely documented.

Deep sequencing technologies have been successfully used to investigate genome-wide polymorphism in various fungi, highlighting the importance of genome organization for pathogen evolution and eventually leading to the proposition of candidate genes implicated in phenotype variations [32–40]. In the case of *F. graminearum*, an annotated genome of reference is available, based on the sequencings of a North-American isolate [41–43]. The latest version consists of 38 Mb distributed in four scaffolds assigned to the four expected chromosomes and has been predicted to contain 14,160 nuclear protein coding genes [41]. The function of the majority of these genes remains unknown [41]. Nevertheless, specific efforts of manually curated genome-mining coupled to proteomics and transcriptomics studies revealed a large arsenal of potential effectors, including potential secreted proteins or secondary metabolites other than the currently known mycotoxins [41, 44–48]. Concerning genome-wide diversity, the first insights have been given after re-sequencing of a second North American isolate at 0.4X, identifying more than 10,000 SNPs located preferentially in chromosomes ends and inner chromosomal locations [42]. Although partial, this first re-sequencing gave a preliminary picture of the organization of the polymorphism in the genome [42]. However, several unanswered questions remained. What are the patterns of polymorphism in the regions of the reference genome not covered by reads produced after re-sequencing? Is this genomic organization respected across worldwide isolates? What is the state of the diversity affecting the functional part of the genome, including the genes for which a role for adaptation could be assumed? In order to answer those questions we proposed to re-sequence six strains of *F. graminearum* originally isolated from various locations in France. These strains all belong to the DON/15-ADON chemotypes, respecting the overrepresentation of this chemotype from French cultivated wheat [20].

The first objective of our analysis is therefore to quantify the whole genomic diversity of French isolates compared to the reference genome. The second objective is to evaluate the potential contribution of this diversity for phenotypic diversity by a systematic variant annotation and an estimation of the encoding-effects for variants located within genes; with a special attention on genes potentially implicated, or previously suggested to be implicated for host-pathogen interaction. By doing so, we were able to conduct a multi-scaled analysis, highlighting the organization of polymorphism in a genome-wide manner and giving access to candidate and individual gene information. Overall, these results strengthen the idea that genome organization plays a major role in the evolution of this pathogen while establishing a solid resource for further targeted genomic and genetic investigations.

## Results

### SNPs and InDels discovery

Our strategy of genome re-sequencing applied to six *F. graminearum* strains generated a total of 125 million of read pairs of 100 base pairs (bp) in length, corresponding to 37.0–44.7 million raw reads per genome (Additional file 1: Table S1). Quality trimming and filtering of reads resulted in 35.5–42.9 million paired-end reads per genome with an average read length of 91 bp. Between 88.4% and 94.8% of these reads were aligned correctly on the reference genome – a total genome coverage of 98.8% (considering all reads produced, 99% for mitochondrial genomes) and sequencing depths ranging from 79.5 X to 93.2 X depending on the considered isolate (Additional file 1: Table S1 and Figure S1). Only 13 protein coding genes of the 14,160 described in the reference nuclear genome were not covered by read in any of the isolate genomes presented herein (Additional file 2). The majority of these genes are located in genomic regions (1 kb upstream and 1 kb downstream) exhibiting deficiency in genome coverage (Additional file 2). Amplification of those targeted genes suggested that those genes are actually absent from the 6 genomes (data not shown). All of these 13 genes were discarded for downstream analysis.

The locations of genetic variations were investigated (Table 1). Variants were called on the basis of a variation compared to the sequence of the reference genome (RRES v4.0). Variant calling was fine-tuned to detect preferentially short size variants, i.e.*,* Single Nucleotide Polymorphisms (SNPs) and short Insertions or Deletions (InDels), and obtained a final dataset of 242,756 highly-confident variants, all strains considered, consisting of 234,151 SNPs (96%) and 8,605 InDels (Table 1, Additional file 3). Regarding the insertion and deletion events, 52% and 50% of them, respectively, concerned single nucleotide positions. The largest insertion is 25 nucleotide-long and the largest deletion is 36 nucleotide-long, with mean lengths for both events being 2.8 bp and -2.7 bp respectively (Additional file 4: Figure S2).

**Table 1:** Variant calling statistics, considering strain-specific reads and considering total reads produced

Isolate	INRA-156	INRA-159	INRA-164	INRA-171	INRA-181	INRA-195	Total^a^
% of reference genome callable^b^	97.7%	97.7%	97.6%	97.6%	97.5%	97.7%	98.3%
Number of SNPs	144,679	146,849	144,802	143,283	145,071	145,840	234,151
Number of InDels	4,929	5,073	4,953	4,844	4,938	4,970	8,605
Total number of variants	149,608	151,922	149,755	148,127	150,009	150,810	242,756
Mean depth of sequencing at variant position (X)	72.3	83.5	78.0	79.5	79.6	80.3	78.9
Mean variant density (variants per kb)	3.9	4.0	3.9	3.9	3.9	4.0	6.4
Number of exonic variants	70,850	71,488	70,259	69,130	70,659	71,676	111,975
Number of intronic variants	10,779	10,821	10,509	10,320	10,682	10,786	17,095
Number of non-genic variants	67,979	69,613	68,987	68,677	68,668	68,348	113,686
Number of variants with French genomes	66,726	69,040	66,873	65,245	67,127	67,928	159,874

^a^: considering all reads produced by whole genome sequencing of the six isolates

^b^: exluding the end of the chromosome IV from 7,953,943 bp onwards, corresponding to repeated RNA encoding sequence (see Methods)

The number of variants per strain ranges from 143,283–146,849 for SNPs, and from 4,844–5,073 for InDels (Table 1). Among them, 82,882 variants (34.1%) are common between all six French isolates. For simplicity purposes, this particular subset of variants will be referred to as the “common block of diversity”. Beside this baseline of diversity, each isolate differs from the other five French isolates by 67,157 genetic variations in average (65,157–69,040; Table 1). Pairwise comparison of isolates shows that INRA-156, with an average of 69,165 variants with each other French isolates, has the most polymorphic genome whereas the genomes of INRA-164 and INRA-181 are the least different with 35,153 variants identified (Table 2). Among the complete set of variable loci identified in this analysis, 1,235 (0.5%) presented different alleles between French alleles, all different that the reference one (i.e. multi-allelic variants).

**Table 2:** Genome-wide comparison of variants between pairs of isolates

Strains	PH-1	INRA-156	INRA-159	INRA-164	INRA-171	INRA-181	INRA-195
PH-1	-	149,608	151,922	149,755	148,127	150,009	150,809
INRA-156	61.6%	-	70,856	66,763	65,709	63,758	78,739
INRA-159	62.6%	29.2%	-	48,501	55,771	62,330	71,769
INRA-164	61.7%	27.5%	20.0%	-	48,453	35,153	68,191
INRA-171	61.0%	27.1%	23.0%	20.0%	-	36,719	66,139
INRA-181	61.8%	26.3%	25.7%	14.5%	15.1%	-	65,433
INRA-195	62.1%	32.4%	29.6%	28.1%	27.2%	27.0%	-

Upper diagonal considers number of variants by pair, lower diagonal considers the part of the overall diversity (242,756 variants) in percent explained by this pair

### Genomic distribution of variants

**Fig. 1:**
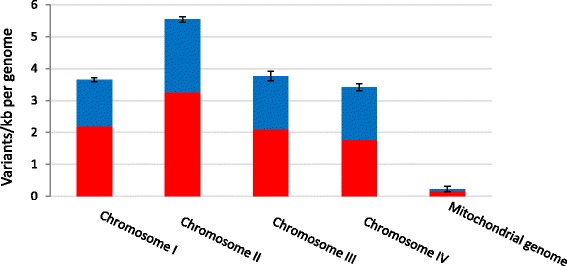
Average variant density by strain for the four chromosomes and the mitochondrial genome. Variant density is represented in variants/kb. The density of variants belonging to the common block of diversity (observed in all French isolates) is in *red*; the density of variant belonging to the diversity observed between French isolates is in *blue*

**Fig. 2:**
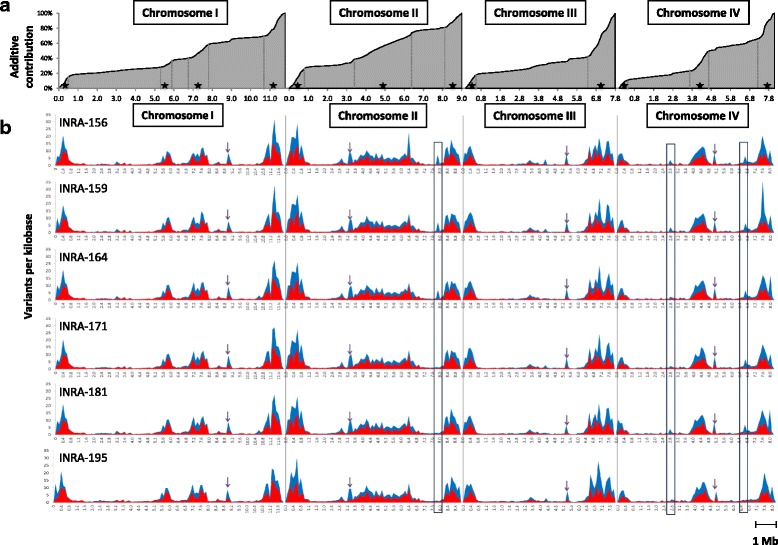
Profiles of variant distribution by chromosome. Density profiles were computed for non-overlapping 100 kb-long sliding windows along the four chromosomes of *F. graminearum*. **a** Cumulative variant density profiles, all polymorphism considered. Star-containing intervals delineated by dotted lines indicate polymorphic islands. **b** Variant density profiles along the four chromosomes of *F. graminearum* for each strain. The density of variants belonging to the common block of diversity (observed in all French isolates) is in *red*; the density of variant belonging to the diversity observed with other French isolates is in *blue. Black* rectangles highlight selected differences between isolates. The arrows indicate the positions of centromeres

Variant average genome-wide density reached 6.6 variants per kilobase (kb) considering the all genomes, ranging from 3.9 to 4.0 variants per kb per individual genome (Table 1). The distribution of the variants is not uniform between and within chromosome. At the inter-chromosomal level, Chromosome II, with 5.4–5.6 variants per kb per genome always exhibits the greatest variant density (Fig. 1). The number of variants detected in the mitochondrial genomes dropped considerably (less than 0.3 variant per kb) compared to nuclear genomes, all variants being localized outside of annotated genic sequences (Additional file 3: Table S3). At the intra-chromosomal level, the contribution of chromosome segments to the overall polymorphism is not linear (Fig. 2a). Telomeric/subtelomeric ends and discrete interspersed interstitial regions participate actively to the total polymorphism. Polymorphic islands are distinguished easily (Fig. 2a, delimited by dot lines and dark stars; accounted for when longer than 200 kb and showing at least a two-fold increase in variant density compared to the genome-wide median density). Such regions present in average a 7.5-fold increase of variant density compared to others (16.0 variants/kb vs. 2.1 variants/kb). The additive length of these regions represents 31.5% of total nuclear genome length while containing 76.7% of the total polymorphism (Additional file 5: Table S4). The presence of polymorphic islands at both chromosome ends are a common feature between chromosomes, whereas the number and size of interstitial polymorphic regions differ: for example, chromosome I exhibits two distinct variant-rich regions, chromosome II has a long continuous variant-rich region spreading over one third of total chromosomic size, chromosome IV displays a single ~1 Mb-long variant-rich region, and chromosome III has none (Fig. 2a, b). The predicted positions of centromeres [41] also appear to collocate with variant-rich regions (Fig. 2b), whereas too short in length to be accounted for polymorphic islands. Variant density is not uniform within polymorphic islands either (Fig. 2b). General variant density profiles are conserved between genomes (Fig. 2b); and between the common block of diversity and the diversity recorded between French isolates (Fig. 2b). This tendency does not exclude occasional differences observed between strains (examples delimited by black rectangles, Fig. 2b). For instance, the region ranging from 7.8 Mb to 8 Mb on chromosome II is rich in variants in the genomes of INRA-156, INRA-159 and INRA-164 but not in those of the other three strains.

### Functional annotation of variants

**Fig. 3:**
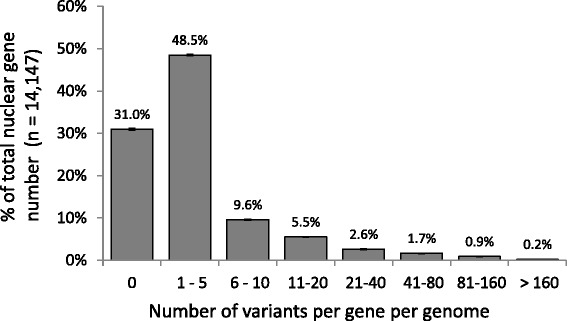
Distribution of average variant content per gene per genome. Values are expressed in percent of total nuclear protein encoding gene number (*n* = 14,147). Bars are mean values for the count of variant considered and error bars the standing deviations per genome

All strains considered, 129,070 variants are found within genic (introns and exons) sequences and 113,686 variants are found elsewhere in the genome (Table 1). Although significant due to the large number of genes, variant density observed within genic sequences does not appear to be greatly reduced compared to the variant density of other sequences (1.05-fold; *p-*value < 0.001). Intronic variants (total: 17,095; per genome: 10,320–10,821) are overrepresented by 5.3-fold (*p-*value < 0.001) whereas exonic variants (total: 111,975; per genome: 69,130–71,676) are slightly underrepresented by 0.9-fold (*p-*value < 0.001). Considering all protein-coding nuclear genes (*n* = 14,147 excluding not covered genes), 80% present at least one mutation in at least one isolate - 69% of genes in average when strains are considered individually (Fig. 3). Median number of variants per gene per genome is 1, whereas the distribution of variant number per gene is skewed due to extreme variant content exhibited by a small percent of genes (Fig. 3).

**Fig. 4:**
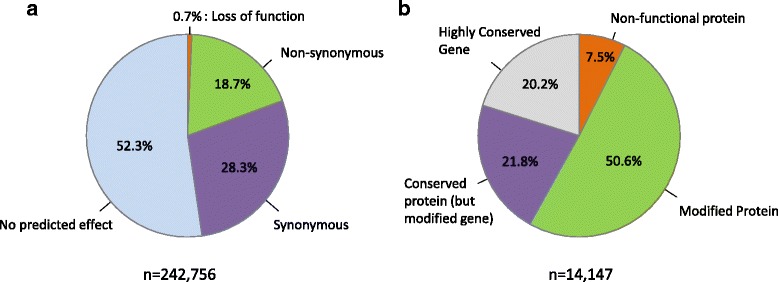
Variant effect prediction and subsequent gene classification. **a** Classification of variants according to their predicted effects (*n* = 242,756). *Orange*: variants leading to a loss of function (LoF) of the proteins; *Green*: variants with non-synonymous effects (including intronic and exonic variants); *Purple*: variants with no predicted effect; *Blue*: variants located outside of genic sequences. **b** Classification of genes according to the type of variant (predicted effect) they contain. Orange: genes containing at least variant(s) leading to a loss of function (LoF) of the proteins; *Green*: genes containing at least variants with non-synonymous effects (including intronic and exonic variants, and containing no LoF variant); *Purple*: genes containing only variants with no predicted effect; *Blue*: genes of which no variants have been detected

In order to identify biological functions possibly more affected than others by variants, we estimated the consequences of genic variants in all strains considered (including introns and exons; Fig. 4a and Additional file 3: Table S3). A little more than half of the variants (52.3%) are predicted to not change protein sequences because they are located in intergenic and intronic regions, outside of splicing sites. Another 28.3% have synonymous effects (i.e.*,* a codon exchange leading to no change in amino acid), 0.7% of total variants have a predicted loss-of-function effect (LoF, in our case the introduction of a frameshift, a stop codon, the loss of the codon start or a critical mutation within the splicing-site), 18.7% have a non-synonymous effect (i.e.*,* a codon exchange leading to a change in amino acid). Genes can also be organized according to their content in variants and their predicted effects (Fig. 4b and Additional file 6: Table S5). Four categories can be defined: the “non-functional” category consists of the 1,057 genes (7.5% of the protein-coding genes) that contain at least variants predicted to lead to a loss of function in at least one isolate; the “Modified Protein” and “Conserved Protein” categories includes 7,164 genes (50.6% of the protein-coding genes) with non-synonymous variant(s) and 3,085 genes (21.8% of the protein-coding genes) with synonymous variant(s) respectively; finally the “Highly Conserved Gene” category (Additional file 6: Table S5) includes genes with no variant identified in any of the isolates (*n* = 2,841, 20.1% of the protein-coding genes).

### Biological functions that can be affected by genetic variants

We investigated the putative functions of the genes belonging to the different categories described above. A gene ontology (GO) term enrichment approach was used to discover top functions represented in gene lists belonging to each category. Results are summarized in Table 3. Category “Non-functional” is significantly enriched in genes implicated in chitin catabolism; category “Modified Protein” is enriched in genes involved in the regulation of transcription, in oxidation and reduction processes and in the regulation of primary metabolic process; category “Conserved Protein” is enriched in genes acting in signalization and communication, translation, protein transport and several process involved for example in carbohydrate metabolism; finally, the “Highly Conserved Gene” category is enriched in genes involved in more universal cellular process, such as cytoplasmic transport including Golgi vesicle transport, protein folding and macromolecule assemblies, translation, as well as several biosynthetic and catabolic processes (Table 3). GO term enrichment analyses are however prone to ontology mapping-related biases [49]. Forty five percent of the totality of nuclear protein-coding genes of *F. graminearum* lack GO term annotation [41]. Therefore, we developed a second approach that consist in using *F. graminearum*-specific gene lists compiled from transcriptomic experiments and genome-mining efforts and available from the literature: transcriptomic data from *in planta* experiment, genes coding for putative secreted proteins, genes belonging to predicted secondary metabolite clusters [41, 48, 50].

**Table 3:** Significant (*p*-value < 0.01) gene ontology enrichment of the categories built from their variant contents and downstream coding-effect

Genes Lists	GO ID	GO Term	Genes in the GO list	Theoretical gene number	Observed gene number	Fold enrichment
"Non-functional"	GO:0006030	chitin metabolic process	21	1	5	4.9
"Modified Protein"	GO:0006355	regulation of transcription, DNA-dependent	458	239	294	1.2
	GO:0055114	oxidation-reduction process	763	398	478	1.2
	GO:0060255	regulation of macromolecule metabolic process	482	250	300	1.2
	GO:0080090	regulation of primary metabolic process	497	258	307	1.2
“Conserved protein”	GO:0044262	cellular carbohydrate metabolic process	30	8	17	2.2
	GO:0007264	small GTPase mediated signal transduction	43	11	22	2.0
	GO:0015031	protein transport	121	31	59	1.9
	GO:0044723	single-organism carbohydrate metabolic process	82	21	36	1.7
	GO:0072521	purine-containing compound metabolic process	90	23	38	1.6
	GO:0006412	translation	181	47	76	1.6
	GO:0007154	cell communication	140	36	56	1.5
	GO:0044267	cellular protein metabolic process	509	132	179	1.4
"Highly conserved genes"	GO:0006888	ER to Golgi vesicle-mediated transport	8	1	6	4.5
	GO:0048193	Golgi vesicle transport	15	3	10	4.0
	GO:0016482	cytoplasmic transport	26	4	12	2.8
	GO:0034622	cellular macromolecular complex assembly	25	4	11	2.6
	GO:0006457	protein folding	40	7	16	2.4
	GO:0022607	cellular component assembly	35	6	14	2.4
	GO:0044283	small molecule biosynthetic process	132	22	46	2.1
	GO:1901136	carbohydrate derivative catabolic process	53	9	18	2.0
	GO:0046394	carboxylic acid biosynthetic process	110	18	37	2.0
	GO:0006996	organelle organization	66	11	22	2.0
	GO:0006412	translation	181	30	59	1.9
	GO:0008652	cellular amino acid biosynthetic process	86	14	27	1.9
	GO:1901565	organonitrogen compound catabolic process	75	13	23	1.8
	GO:0071840	cellular component organization or biogenesis	134	22	39	1.7
	GO:1901566	organonitrogen compound biosynthetic process	190	32	54	1.7
	GO:0044267	cellular protein metabolic process	509	85	128	1.5
	GO:0044281	small molecule metabolic process	463	77	110	1.4
	GO:1901564	organonitrogen compound metabolic process	400	67	95	1.4
	GO:0044249	cellular biosynthetic process	788	132	174	1.3
	GO:0019538	protein metabolic process	680	114	141	1.2

**Fig. 5:**
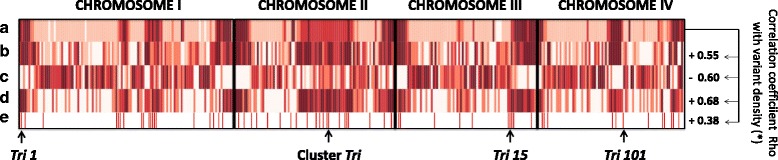
Heatmap representation of variant and gene counts per 100 kb-long non-overlapping windows. Spearman rank order correlation coefficients were computed between variant and gene counts. The star * indicates that all correlations are significant at the threshold *p* = 0.01. A. Genetic variants (*n* = 242,756). B. Host-specific genes (*n* = 2,353) [50]. C. *In planta*-constitutive genes (*n* = 5,029) [50]. D. Secreted protein-encoding genes (*n* = 616) [41]. E. Secondary metabolite-encoding gene clusters (*n* = 67) [48]. The positions of the *Tri* cluster and the not-clustered *Tri* genes *Tri1*, *Tri15* and *Tri101* are indicated by arrows

**Fig. 6:**
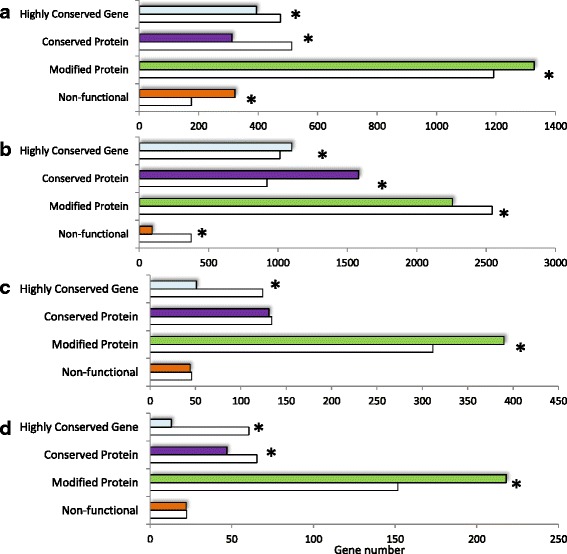
Selected *F. graminearum*-specific gene content of each category of predicted variant effect. For each category, actual gene counts (colored bars) are compared to the theoretical counts expected under hypothesis of random distribution of variants (*white*). The star * means Chi-squared test was significant (*p-*value < 0.001). **a** Host-specific genes (*n* = 2,353) [50]. **b** *In planta*-constitutive genes (*n* = 5,029) [50]. **c** Secreted protein-encoding genes (*n* = 616) [41]. **d** Clustered secondary metabolite-encoding gene (*n* = 301)

The first list derives from *in planta* transcriptomic experiments that identified genes showing unique host-specificity of expression (17% of total nuclear gene number, *n* = 2,353) by contrast with genes showing constitutive expression (36% of total nuclear gene number *n* = 5,029) suggested to correspond to basal and universal mechanism of host infection ([50], Additional file 6: Table S5). We observed a positive correlation between locations of polymorphisms and location of host-specific genes (Spearman rank order Rho = 0.55, Fig. 5 lane B). Host-specific genes are found overrepresented in the categories “Non-functional” and “Modified Protein” and underrepresented in the categories “Conserved Protein” and “Highly Conserved Gene” (Fig. 6a). This observation suggests than non-synonymous mutations tend to be accumulated into these genes. Indeed, loss-of-function and non-synonymous variants are particularly found within these genes with a 2.1-fold and 1.8-fold enrichment, respectively (Additional file 7). Conversely, the locations of genes expressed constitutively in all *in planta* conditions is negatively correlated to the locations of variants (Rho = - 0.60, Fig. 5 lane C). These genes are overrepresented in the categories “Highly Conserved Gene” and “Conserved Protein”, and underrepresented in the categories “Modified Protein” and “Non-functional” (Fig. 6b). Similarly, these genes contain less loss-of-function and other non-synonymous variants (5.6 times and 2.5 times respectively; Additional file 7).

The second list consists of genes with typical motifs suggesting that they code for secreted proteins that could therefore be potential effectors (*n* = 616; 126 have been shown to be expressed in a host-specific manner). The spatial distribution of these genes positively correlates with the genome-wide distribution of polymorphisms (Rho = 0.68, Fig. 5 lane D). These secreted protein-encoding genes are found overrepresented in the category “Modified Protein” and underrepresented in the category “Highly Conserved Gene” by 1.25 and 0.41-fold respectively (Fig. 6c). These genes are further enriched in non-synonymous mutations (other than loss-of-function) by 1.38 fold (Additional file 7).

### Focus on secondary metabolites clusters and TCTB biosynthetic genes

Finally, we investigated genes predicted to be implicated in the biosynthesis of secondary metabolites and (mostly) organized in clusters on the genome (*n* = 301). The genomic distribution of these genes is significantly correlated with polymorphism (Rho = 0.38, Fig. 5 lane E). They are significantly overrepresented in the category “Modified Protein” and significantly underrepresented in the categories “Highly Conserved Gene” and “Conserved Protein” (Fig. 6d). These genes are indeed enriched in non-synonymous variants, but show in the other hand a reduction of LoF mutations (Additional file 7 and Additional file 8: Table S6). Still, 24 genes belonging to 20 different secondary metabolite clusters are affected by LoF variant(s) in at least one isolate (Additional file 8: Table S6). This is the case for example of the gene FGRRES_15980_M, coding a probable polyketide synthase involved in zearalenone biosynthesis, which contains a conserved loss of function variant in all French isolates (Additional file 8: Table S8). Remarkable secondary metabolites are the type B trichothecenes (TCTB), including the deoxynivalenol (DON), reported to be involved in pathogenicity [51]. We examined the polymorphisms affecting *Tri* genes (*n* = 15) involved in the biosynthesis of TCTB (12 of them are clustered on chromosome II as indicated on Fig. 5; Additional file 8: Table S7). An overall of 252 variants have been identified within the genic sequences and the intergenic sequences of *Tri* genes (located in the upstream and downstream sequences for the non-clustered *Tri* genes; Additional file 8: Table S8). Among these variants, 131 belong to the common block of diversity (observed in all six genomes analyzed herein). Only four of the rest of the variants are predicted to bring non-synonymous effects other than loss-of-function. All of them are located within the coding sequence of *Tri15* and affect the strains INRA-159, INRA-164, INRA-171 and INRA-181 (Additional file 8: Table S7). *Tri15* gene encodes a putative transcription factor and does not seem to be implicated in TCTB production [5].

### Genes showing an excess of non-synonymous effect mutations

In order to identify genes accumulating non-synonymous effect mutations, we consider the total polymorphism detected in this analysis and extracted 797 genes that accumulated either or both non-synonymous (NS) and LoF mutations (NS + LoF > total number of mutation, minimum total number of mutation = 4; Additional file 6: Table S5). The large majority of them (64%) is located within polymorphic islands (Additional file 6: Table S5). Twenty-nine of them have been previously shown to be both expressed *in planta* and predicted to be secreted (Table 4). Fifteen have been shown to be expressed in a host-specific manner and only one has been shown to be expressed constitutively in all *planta* conditions tested (Table 4). Remarkably, all of them have no known function according to reference genome annotation [41], with the exception of FGRRES_04689 that code for a rhamnogalacturonase A, involved in cell wall polysaccharide degradation. Seven of them contain LoF variants (FGRRES_16333, FGRRES_03521, FGRRES_12210, FGRRES_04646_M, FGRRES_13876, FGRRES_07699, and FGRRES_09118). For FGRRES_04646_M, the mutation is present in every French isolates tested. This gene is unlikely to be an essential effector during infection of wheat as several strains of this sample have been shown to be highly aggressive (Table 5; Additional file 3: Table S3). In the other hand, the gene FGRRES_07699 is predicted to be non-functional in the highly aggressive strain INRA-156 only; the gene FGRRES_12210 is predicted to be non-functional in the less aggressive strain INRA-195 only. These genes represent interesting effectors that could have escaped from the host defense for the first case or implicated in aggressiveness reduction for the second case. The knowledge on the diversity of these genes might help further investigations.

**Table 4:** Putative effectors showing an excess of non-synonymous effect mutations

Ensembl gene ID	FGSG	Chrom	Gene start (bp)	Gene end (bp)	Gene description	InterPro ID	InterPro short description	Homology	Protein length	% cysteine	variants LoF	variants Non-Synonymous	Total variants^a^	Ratio of non-synonymous effect mutations	Polymorphic Islands	*In Planta* Expression [50]
FGRRES_11675	FGSG_11675	I	356,230	357,118	Uncharacterized protein	-	-	-	279	1.07	0	7	10	0.7	Yes	Wheat
FGRRES_01778	FGSG_01778	I	5,860,579	5,861,567	Uncharacterized protein	-	-	-	277	2.16	0	5	9	0.6	Yes	Wheat
FGRRES_02228	FGSG_02228	I	7,225,618	7,227,797	Uncharacterized protein	IPR000120, IPR023631	Amidase	glutamyl-trna amidotransferase subunit a [Fusarium langsethiae]	664	0.75	0	10	19	0.5	Yes	Wheat & Barley
FGRRES_02269	FGSG_02269	I	7,357,559	7,358,332	Uncharacterized protein	-	-	-	237	2.1	0	4	7	0.6	Yes	Wheat & Barley
FGRRES_13692	FGSG_13692	I	9,626,040	9,628,066	Uncharacterized protein	-	-	-	499	1.6	0	4	7	0.6	No	Wheat & Barley
FGRRES_07993	FGSG_07993	II	110,904	113,251	Uncharacterized protein	IPR001764, IPR002772, IPR017853, IPR026891, IPR026892	Glycoside hydrolase/Fn3 like	exo- -beta-xylosidase bxlb [F. langsethiae]	766	1.3	0	5	7	0.7	Yes	Wheat & Barley
FGRRES_17022	-	II	1,652,257	1,656,378	Uncharacterized protein	-	-	muc1-extracellular alpha- -glucan glucosidase [F. langsethiae]	1340	0.3	0	5	6	0.8	No	Wheat
FGRRES_16333	-	II	4,194,219	4,196,939	Uncharacterized protein	-	-	-	887	3.27	3	41	80	0.6	Yes	Barley
FGRRES_03274	FGSG_03274	II	4,695,334	4,698,042	Uncharacterized protein	IPR029167	Mug117	-	884	1.92	0	12	22	0.5	Yes	Wheat & Barley
FGRRES_03521	FGSG_03521	II	5,366,512	5,367,123	Uncharacterized protein	IPR009327, IPR011051, IPR014710	RmlC-like cupin domain	putative cupin family protein [Diaporthe ampelina]	184	1.62	1	5	7	0.9	Yes	Wheat
FGRRES_03612	FGSG_03612	II	5,604,284	5,605,254	Uncharacterized protein	IPR001087, IPR013830	Lipase_GDSL, SGNH hydrolase-type esterase domain	gdsl lipase acylhydrolase [F. langsethiae]	289	1.72	0	4	7	0.6	Yes	Wheat & Barley
FGRRES_12405_M	FGSG_12405	II	5,622,275	5,622,943	Uncharacterized protein	IPR003609	Pan_apple	-	222	4.04	0	8	9	0.9	Yes	Wheat & Barley
FGRRES_03944	FGSG_03944	II	6,465,510	6,466,808	Uncharacterized protein	IPR011042	Six-bladed beta-propeller, TolB-like	serum paraoxonase arylesterase [F. langsethiae]	432	0.46	0	5	8	0.6	Yes	Wheat & Barley
FGRRES_03972	FGSG_03972	II	6,548,953	6,550,914	Uncharacterized protein	IPR006094, IPR012951, IPR016166, IPR016169	flavin adenine dinucleotide linked oxydase; Berberine & berberine-like; CO dehydrogenase flavoprotein-like	6-hydroxy-d-nicotine oxidase [F. langsethiae]	585	1.54	0	7	10	0.7	Yes	Barley
FGRRES_04429	FGSG_04429	II	7,989,077	7,992,064	Uncharacterized protein	IPR003609	Pan_apple	-	995	5.22	0	4	7	0.6	No	Wheat
FGRRES_12210	FGSG_12210	II	8,620,515	8,622,358	Uncharacterized protein	IPR003609	Pan_apple	-	596	2.18	1	10	18	0.6	Yes	Wheat
FGRRES_04646_M	FGSG_04646	II	8,655,498	8,656,180	Uncharacterized protein	-	-	-	225	2.65	2	12	22	0.6	Yes	Wheat
FGRRES_04689	FGSG_04689	II	8,765,660	8,767,148	Rhamnogalacturonase A	IPR000743, IPR011050, IPR012334	Glycoside hydrolase, family 28; Pectin lyase	probable rhamnogalacturonase A precursor [Fusarium fujikuroi IMI 58289]	446	2.46	0	7	13	0.5	Yes	Wheat & Barley
FGRRES_05719	FGSG_05719	III	3,177,333	3,180,794	Uncharacterized protein	IPR029167	Meiotically up-regulated gene 117 protein	-	1153	1.73	0	4	6	0.7	No	Wheat & Barley
FGRRES_05847	FGSG_05847	III	3,549,004	3,550,475	Uncharacterized protein	IPR001002, IPR002509, IPR011330, IPR018371	Glycoside hydrolase/deacetylase, beta/alpha-barre; Chitin-binding, type 1	bifunctional xylanase deacetylase [F. langsethiae]	454	5.71	0	3	5	0.6	No	Wheat & Barley & Maize
FGRRES_12835	FGSG_12835	III	3,658,250	3,659,015	Uncharacterized protein	-	-	-	162	2.45	0	4	7	0.6	No	Wheat
FGRRES_16623	-	III	4,751,122	4,755,561	Uncharacterized protein	IPR013830	SGNH hydrolase-type esterase domain	chitinase [Fusarium oxysporum Fo47]	1461	1.92	0	6	11	0.5	No	Wheat
FGRRES_13876	FGSG_13876	III	6,370,523	6,371,374	Uncharacterized protein	IPR013781	glycosyl hydrolase catalytic domain	Glycoside hydrolase, superfamily [Cordyceps confragosa RCEF 1005]	283	1.41	3	18	40	0.5	Yes	Wheat
FGRRES_17469	FGSG_13850/FGSG_13851	III	6,711,891	6,714,195	Uncharacterized protein	-	-	related to DAN4-Cell wall mannoprotein [Fusarium proliferatum]	719	1.53	0	7	12	0.6	Yes	Barley
FGRRES_11379	FGSG_11379	III	7,412,583	7,413,959	Uncharacterized protein	-	-	-	458	1.96	0	12	23	0.5	Yes	Wheat
FGRRES_06610	FGSG_06610	IV	582,670	585,011	Uncharacterized protein	IPR018946, IPR029052, IPR032093	Alkaline phosphatase D-related	alkaline phosphatase D precursor [Fusarium fujikuroi]	631	0.79	0	5	9	0.6	No	Wheat & Barley
FGRRES_07686_M	FGSG_07686	IV	4,196,885	4,197,838	Uncharacterized protein	-	-	activator of stress protein 1 [F. langsethiae]	317	2.2	0	8	14	0.6	Yes	Wheat & Barley
FGRRES_07699	FGSG_07699	IV	4,231,666	4,232,190	Uncharacterized protein	-	-	-	174	2.29	1	5	10	0.6	Yes	Wheat & Barley
FGRRES_09118	FGSG_09118	IV	7,533,260	7,536,888	Uncharacterized protein	IPR001002, IPR001223, IPR011583, IPR013781, IPR017853, IPR018371, IPR029070	Chitin binding; chitinase II	related to chitinase [Fusarium proliferatum]	1159	3.62	3	10	21	0.6	Yes	Wheat

^a^ total variant numbers include variant detected within the 100 base pairs located in upstream and downstream of the genic sequences

## Discussion

The presented work examines the level of variation that can be observed between the genomes of different *F. graminearum* isolates at the sequence level. In addition to describing genome-wide polymorphisms, this analysis proposes, for the first time, to quantify the downstream effects of the observed variants, particularly exonic as well as intronic variants that can lead to important consequences on the translation product [52]. Here, we applied a whole-genome reference-based DNA re-sequencing strategy rather than *de novo* assembly previously described as more sensitive to sequencing errors [53]. Using a re-sequencing method, the accuracy of variant calling greatly depends on the quality of the read alignments on the reference genome and the depth of read coverage per base. Filters must be applied to differentiate true variants from sequencing errors while keeping the false negative rate low. There is no « one size fit all » situation and settings must be adjusted according to the type of genetic variant investigated [53]. For *F. graminearum*, stringent filtration is further possible (and recommended) as this fungus is haploid and one allele is expected at the positon. A preliminary test-run indicated that SNPs and short InDels are mostly found in the genome of *F. graminearum* with very few variants of larger sizes (data not shown). Consequently, the filters applied to the analysis presented here were set as optimal for SNPs and short InDels detection for enhanced variant discovery.

### *F. graminearum* genome-wide polymorphism is consistent with its pathogenic lifestyle

The comparison of six genomes of French isolates with the reference genome of the PH-1 strain of *F. graminearum* [41] produced a highly confident set of 242,756 distinct variants total. Each of the six genomes presented an average of 150,039 variations when compared to the PH-1 reference genome, and ~ 67,157 variations when compared to each other. This number is much higher than the 10,495 SNPs identified in the first published investigation of the genome-wide polymorphism between another North-American isolate and the PH-1 strain [42]. The much lower number of variants then observed is certainly linked to a very low fold-coverage, 0.4X, being insufficient for exhaustive and confident variant calling [42]. More recently, Walkowiak et al. [54] assembled two genomes of *F. graminearum* representative of the two chemotype-based populations observed in Canada, DON/3-ADON and DON/15-ADON. They reported 147,555 and 103,774 SNPs with the reference genome respectively, as well as 148,978 SNPs between the two Canadian isolates, approximating the level of polymorphism reported herein. Altogether, an estimate of ~150,000 SNPs seem to be a typical variant content expected to be observed between genomes of geographically distal isolates or belonging to different populations. Along the same line, the reduced level of polymorphism observed between French isolates may suggest that these strains are likely to belong to the same population.

On a broader scale, the genome-wide level of polymorphism observed between isolates of *F. graminearum* (~4 SNP/ Kb) is consistent with levels of polymorphism exhibited by other pathogenic fungi, as reported in the causal agent of poplar rust *Melampsora larici-populina* (~2 SNP/Kb; [37]), the causal agent of the wheat stripe rust *Puccinia striiformis* (~5 SNPs per Kb; [55]) or in the human pathogen *Coccidioides immitis* (~5 SNP/Kb); [56]). These levels are lower than the one revealed between isolates of *Botrytis cinerea* that could reach 10 variants per kb [38]. Authors suggested that such genome-wide diversity is linked to the ability of this pathogen to infect a remarkably broad range of hosts. In comparison, the human genome exhibits between 1.2 and 1.5 variants per kb [57]. The higher values observed in fungal pathogen genomes may be a consequence from their parasitic lifestyle that pushes their need to evolution up [58]. According to the criteria given by McDonald and Linde [23] to estimate the potential of evolution of fungal pathogen, *F. graminearum* can be considered as a high-adaptive potential pathogen. Paradoxically, the various analyses aiming at identifying parts of the genome that are under selection for diversification failed to identify genomic regions under strong selection [11, 59]. In the present analysis, we use total genome information and identify 797 candidate genes accumulating missense and nonsense mutations. The functions of these genes are unknown for the majority but their potential implication in pathogenicity and adaptation certainly calls for in-depth investigations.

### The multi-scaled location of polymorphisms in the genome

The genome-wide average value of variant density is not a metric sufficient enough to fully comprehend the patterns of polymorphism in *F. graminearum*; indeed it does not describe the remarkable discrete variations that we observed at both inter- and intra- chromosomal dimensions. For example, chromosome II is more polymorphic than the other chromosomes. This higher speed of diversification of this individual chromosome has been previously reported in genome-wide comparisons of close species of the *Fusarium* genus [59]. The authors hypothesized that the chromosome II of *F. graminearum* could play a preponderant role for host infection and adaptation [59, 60]. Our analysis is in line with such a hypothesis. Distribution of polymorphisms is also highly uneven within each chromosome. The single-base resolution of our analysis enabled the accurate definition of polymorphism islands along chromosomes. Polymorphisms are preferentially located at the ends of chromosomes. Such phenomenon is common in fungal pathogens and more generally in eukaryotic genomes [58]. The interstitial polymorphic islands are, for their part, more original. These regions have been previously investigated and are described as telomeric-like and subtelomeric-like regions that originate from ancestral chromosomes fusion events [42, 60]. Remarkably, the chromosomal landscapes of polymorphism reported herein follow striking similitude with the lower-coverage analysis reported in 2007 [42]. Considering these patterns of polymorphism highly conserved between isolates, we may hypothesize that genome architecture plays a predominant role in shaping the polymorphism landscape, instead of evolution forces. Several mechanisms have been previously proposed as driving genome structure of fungal pathogens [61], among which the action of meiotic recombination may play an important role through the preferential shuffling of particular chromosome regions [62]. An inconsistency of recombination rate has been already reported along *F. graminearum* genome, and the increases of recombination activity seem to collocate with variant rich regions [42]. These same regions were further showed to be enriched in specific epigenetic mark [63], of which implication for meiotic recombination through chromatin remodeling has never been tested. Thus, the weight of individual contributions and inter-connections between the different proposed elements of regulation remains however unclear [61]. A comprehensive investigation of these different phenomena could shed light on the events driving the organization of *F. graminearum* genome and its evolution.

Finally, we observed that polymorphism rates are highly variable at gene level, with introns being more polymorphic than exons. Such situation could be the result of selective pressures since exonic variants can more directly affect protein function, as such, can be rapidly unselected [64]. Nevertheless, this does not mean that variants located in introns, and typically classified as synonymous mutations, have no contribution in protein polymorphism. Indeed, the demonstration has been done that mutations located in introns can have important effect, notably by altering the splicing process [52, 65]. Accordingly, the presented work takes all variants into consideration during the annotation process [66], identifying 1,647 variants with predicted loss of function effect and 45,196 variants with other predicted non-synonymous effects.

### Further evidence of a two-speed genome organization in *F. graminearum*

Our data reveal a remarkable positive correlation between specific biological functions and polymorphism along the genome. For example, polymorphic islands are enriched in genes with roles in biotic and abiotic adaptation, these genes exhibiting a greater level of polymorphism than genes with basal and vital functions. This result is especially true for genes coding for secreted proteins or belonging to secondary metabolite biosynthesis clusters; both categories of genes that have been suggested to play preponderant roles during pathogenesis [41, 44]. As a whole, plant-specifically expressed genes, which translate host-specific mechanisms of infection [50], are overrepresented in these hotspots of diversity and are more diverse than other genes. In the line of our above hypothesis, we propose that this correlation arise from the preferential location of certain biological functions according to the organization of the polymorphism in the genome rather than a result of the historical and ongoing diversifying selection acting directly on these genes. Such genomic organization could argue in favor of contrasted abilities of evolution of *F. graminearum* gene repertoire - with genes implicated in basal process being placed in conserved compartments and genes with a bigger need of evolution being placed in highly diversifying chromosomal segments.

With the convergence of knowledge about fungal genomes, such “two-speed” organization seems to be a frequent feature in filamentous pathogens [58, 61, 67–70]. Nevertheless, in several pathogenic species, a faster speed of evolution has been attributed to gene sparse, repeat-rich compartment, as in *Leptosphaeria maculans,* or dedicated dispensable chromosomes, as observed in *Zymoseptoria tritici* [61, 67]. Compared to closely related species, as in *Fusarium oxysporum*, no dispensable chromosome has been ever observed in *F. graminearum*. Moreover, its genome is quite compacted and relatively poor in repeated sequences [41, 42]. This decreased number of repeated sequences compared to some other *Fusarium* species has been previously attributed to the action of the repeat induced mutation system (RIP) that introduces point mutations within repeated sequence and therefore protects the genome from the mobility of transposons [42]. Therefore, this system may have participated to the organization of the polymorphism in the genome of *F. graminearum* [42, 61]. Altogether, *F. graminearum* is an original and interesting model for the investigation of genome architecture in evolution as well as improve our understanding about the theory of two-speed genome evolution of fungal pathogens.

### The added-value of genomic data to identify genes involved in pathogenicity

The level of polymorphism affecting gene sequence is remarkable. Indeed, up to 69% of the genes are polymorphic per genome compared to the reference sequence. Such polymorphism should be taken into account for genome editing approaches, often designed from the consensus sequence of the reference genome.

Quantitative variations of mycotoxin production and aggressiveness have been previously observed within field populations of *F. graminearum* [71]. Although variable according to environment, heritability of these traits has been demonstrated and suggests the action of multiple genetic factors [11]. To associate genetic variation with phenotype changes remains however challenging. The polymorphisms of genes involved on TCTB production, as the biosynthetic *Tri* gene cluster, have been previously suggested to be responsible to aggressiveness variation in *F. graminearum* [8]. Likewise, a genome-wide association survey associated variants located in 27 different genes to variations in aggressiveness, all of them unlinked to mycotoxin production [11, 72]. The isolates used in the present survey presented contrasted level of aggressiveness in wheat, correlated to contrasted level of DON production (Table 5). These phenotypic characteristics were further shown to be stable, as repeatable and not dependent to the wheat variety inoculated (data not shown). Although these phenotypes may be related to genetic variation, none of the variants described previously were retrieved in French isolates; and may indicate that the genetic bases of aggressiveness and TCTB production may be more complex. As a preliminary investigation, we separated our sample into two discrete groups according to aggressiveness and DON production and consider the distribution of variants. Therefore, four hundred eighty four genes were conserved in highly toxinogenic and aggressive isolates, and accumulating non-synonymous mutation(s) in less toxinogenic and less aggressive isolates (data not shown). Genes coding for vesicle trafficking were found to be significantly impacted by mutation in the less toxinogenic and less aggressive isolates. This observation is consistent with the vesicle-mediated secretion of TCTB in *F. graminearum* [73], the regulation of secondary metabolism and cellular compartmentalization of biosynthesis pathways being tightly linked in fungi [74].

## Conclusion

Whole-genome sequencing of six *F. graminearum* isolates revealed a remarkable number of polymorphisms, with an overall of 242,756 highly confident variants. Polymorphisms are preferentially found clustered in the genome and may play a role in the diversification of the gene repertoire implicated in host-pathogen interaction. We further hypothesize that fungal biological functions are organized in such a way that they take full advantage of the evolving dichotomy proposed by the intrinsic architecture of this pathogen’s chromosomes. The molecular control of intrinsic chromosome features remains however to be investigated. Our observations further emphasize the high-adaptive potential of this pathogen and defend the use of more integrative pest management. As a whole, this detailed description of the genetic and functional diversity of these genomes is a milestone on the path to dissect the genetic bases of important history-life traits of *F. graminearum*.

## Methods

### Fungal isolates

Six strains of *Fusarium graminearum sensu stricto* were isolated from wheat plants cultivated in several French regions between 2001 and 2002 ([75], Table 5). These strains exhibit various trends of pathogenicity and quantitative profiles of DON/15-ADON production that are representative of the genotypic and phenotypic diversity observed within a larger French collection of isolates ([75], Table 5). PH-1 strain was originally isolated from corn in Michigan (NRRL 31084). The strain has been shown to be highly fertile, produces trichothecenes and zearalenone, sporulates abundantly in pure culture and is highly pathogenic to wheat and barley [76, 77].

**Table 5:** Phenotypic information and geographical origin for the six strains

Strains	TCTB production in vitro	Aggressiveness on wheat	TCTB production in wheat	Location in France/Administrative division
INRA-156	++	+++	+++	Center/Cher (18)
INRA-159	++++	0	0	Center/Cher (18)
INRA-164	++++	++++	++++	North/Seine Maritime (76)
INRA-171	0	++	++	South-West/Gers (32)
INRA-181	++	+++	+++	North/Eure (27)
INRA-195	0	+	+	North-East/Meuse (55)

### Extraction of genomic DNA and sequencing

Genomic DNA was extracted from ~50 mg of lyophilized mycelium previously grown for five days on potato dextrose agar (39 g/l, Difco). Mycelia were lysed in 600 μL of a buffer containing 100 mM Tris-HCl (pH 9.0), 10 mM EDTA, 1% sarkosyl, and proteinase K 200 μg/mL for 2 h at 65 °C. After centrifugation (10 min at 10,000 g), the supernatant was extracted successively with 1 volume of phenol, 1 volume of phenol: chloroform (50:50) and finally 1 volume of chloroform. Nucleic acids were precipitated with 0.1 volume of cold sodium acetate (pH 5.5, 3 M) and two volumes of isopropanol and agitated twice. Solution was then centrifuged 10 min at 10,000 g and supernatant were eluted. DNA precipitate was washed twice with 1 mL cold 70% ethanol for 5 min. After centrifugation, DNA was air dried 5 min using SpeedVac. DNA was dissolved in 100 μL nuclease-free water. Preparation of the libraries and sequencing was performed at the Montpellier GenomiX sequencing platform (France, http://www.mgx.cnrs.fr). Briefly, quantities of genomic DNA were measured using a Qubit® Fluorometer (Life Technologies) and DNA integrity was verified by electrophoresis on Bioanalyzer (Agilent). DNA libraries were prepared from one μg of DNA per strain using TruSeq DNA sample preparation kit (Illumina) following the manufacturer’s instructions for 6-plexed samples (library size 350 bp +/-50 bp). Sequencing was performed on one lane of Illumina HiSeq 2000 generating 100 bp-long paired-end reads. Post-run read quality was verified using FastQC ([78], v0.11.2).

### SNP & InDel discovery and analysis

Reads were cleaned up using PRINSEQ v0.17.1 [79]. Briefly, duplicated reads were removed and the 9th first 5’ nucleotides were systematically trimmed due to skewed base composition introduced by sequencing preparation. Reads with an overall mean Phred-scaled value less than 20 were discarded. Remaining reads were further 3’ trimmed for quality (Phred scale threshold of 20); high quality paired-end reads with length greater than 20 nucleotides were aligned on the genome version RRes V4.0 ([41], [EMBL-EBI accessions HG970331, HG970332, HG970333, HG970334, and HG970335]) using BWA (v0.7.8) and BWA-MEM with standard parameters and a seed size of 15 nucleotides [80]. Invalid paired with aberrant insertion size or unpaired alignments were filtered out using Samtools v 0.1.19 [81]. Valid alignments were annotated using Picard tools (v1.88, http://broadinstitute.github.io/picard/) for further processing with the GATK suite (v2.4, [82]). Identification of genomic regions that could not be mapped or called for variants considering our sequencing set up was conducted using the CallableLoci module of GATK. Alignments on the last 1.38 Mb of the end of chromosome IV systematically showed poor quality scores and were removed from the analysis. This result arises from the large number of rRNA-encoding repetitive sequences units [41] which lengths approximate the length of the sequenced reads resulting in non-unique alignments that are excluded from the analysis. This specific region, however, does not contain known protein-coding genes.

The six BAM files were simultaneously used to discover SNPs and InDels using GATK and the Unified Genotyper walker in haploïd mode [82]. Depth of read coverage per 100 kb-long non-overlapping bins was calculated using the bamCoverage tool of the Bamtools suite (version 1.5.9.1.0). Positions of InDels were recalibrated using the RealignerTargetCreator and the IndelRealigner modules of GATK with standard parameters. A variant was called when observed on at least 80% of the reads aligned at the position with a read coverage more than 5 reads, and a confidence threshold greater than 250 (SelectVariants module of GATK, v2.4). Other parameters were standards. Variant densities for 100 kb-long non-overlapping windows and other statistics on the resulting Variant Call Format (VCF) files were obtained using Vcftools (v0.1.12a, [83]). Regions longer than 200 kb showing at least a two-fold increase in variant density compared to the genome-wide median density where considered as polymorphic islands.

### Functional variant annotation

Genetic variants from specific genomic compartments (i.e.*,* exons, introns, and intergenic regions) were annotated and their effect predicted using snpEff [66] and a custom database constructed from the reference genome (RRes v4.0) and the GTF file associated. Briefly, the software classifies variants found within genes (exons and introns) according to their downstream effect at the protein level (under the standard genetic code). A full list of mutation event classification is available (http://snpeff.sourceforge.net/SnpEff_manual.html). Using this classification, four categories of genes were distinguished: i) the category “Non-functional”, i.e.*,* genes containing variant(s) predicted to lead to a loss of the function of the protein; ii) the category “Modified Protein”, i.e.*,* genes containing only variant(s) predicted to bring one or few amino acid composition changes; iii) the category “Conserved Protein”, i.e.*,* genes containing only variant(s) which do(es) not change amino composition of protein; iv) the category “Highly Conserved Gene”, i.e.*,* genes with no variant detected. Then, we calculated the ratio between the number of mutations with non-synonymous effect (including non-synonymous exonic and intronic variants as well as loss-of-function variants) and the total number of variants within genic sequences (including 100 bp downstream and upstream). Genes were accepted to show an excess of mutation with non-synonymous effect if they contained at least 4 mutations and a ratio of non-synonymous effect mutation on total number of mutation greater than 0.5 (corresponding to a two-fold increase of the genome-wide median number). PROVEAN software [84] was finally used to estimate the functional importance of missense mutations found in genes coding for secreted proteins (as described in King et al., [41]), genes expressed in wheat, barley and/or maize [50], and genes predicted to belong to secondary metabolite pathways (retrieved from Sieber et al., [48]).

### Statistical analyses

Annotation enrichment analysis was conducted using the Gene Ontology Enrichment tools proposed online by the EuPathDB project [43, 85] using Biological ontology and InterPro predictions. Enrichments were accepted for *p*-value lower or equal to 0.01. Chi-squared test was used to compare the observed distribution of the number of genes affected by mutations in the genome with the theoretical distribution of the number genes affected by mutations under the hypothesis of a random distribution of variants in the genome. Similarly, variant enrichment in the polymorphic islands or in genes was tested using the Chi-squared test. Over and underrepresentation were accepted for *p-*value < 0.001. Gene-enrichment in variant-rich region was determined by calculating the density of both variants and genes in non-overlapping 100 kb windows and testing each window for Spearman rank order correlation at the significance threshold of *p-*value < 0.001.

## Additional files

Additional file 1: Table S1. Metrics and statistics of read sequencing and alignment on the reference genome (version RRES V4.0). **Figure S1:** Minimum and maximum read depth (mean number of reads at the position) calculated along sliding 100 kb bin windows of the four chromosomes of the RRES reference genome using the six bam files produced after re-sequencing of the French isolates. (XLSX 49 kb)
Additional file 2: Table S2. Genes described the reference genome (RRES v4.0) not covered by reads in any isolate genomes. (XLSX 16 kb)
Additional file 3: Table S3. Information and annotations for all genetic variants detected in this analysis. (XLSX 32569 kb)
Additional file 4: Figure S2. Distribution of the length of the variants identified in this analysis. Polymorphisms of length equal to zero are SNPs. (JPG 65 kb)
Additional file 5: Table S4. Details of the polymorphic islands delineated during the analysis. (XLSX 15 kb)
Additional file 6: Table S5. Gene annotation according to the polymorphism detected in the analysis. (XLSX 4054 kb)
Additional file 7: Figure S3. Variant enrichment of genes with candidate functions. Representation of the observed variant number (in color) compared to the theoretical number expected under hypothesis of random distribution of variants (white). A. Variants predicted to lead to a loss of function of the protein. B. Variants predicted to have non-synonymous effects on the protein. “*” means Chi-squared test was significant (*p-*value < 0.001). (JPG 57 kb)
Additional file 8: Table S6. Annotation of genes previously predicted to belong to secondary metabolite clusters. **Table S7:** Summary of known *Tri* genes implicated in trichothecene of type B (TCTB) production. **Table S8:** Annotation of *Tri* genes and surrounding sequences according to the polymorphism detected during this analysis. (XLSX 122 kb)

## Abbreviations

15-ADON: 15-acetyldeoxynivalenol
3-ADON: 3-acetyldeoxynivalenol
DON: deoxynivalenol
FHB: Fusarium head blight
GO: Gene ontology
InDel: Insertion or deletion
LoF: loss of function
NIV: nivalenol
NS: non synonymous
SNP: Single nucleotide polymorphism
TCTB: Type B Trichothecenes
VCF: variant call format
