# Supplementary figures and images for: Landscape of genomic diversity and host adaptation in Fusarium graminearum
Source: BMC Genomics. 2017 Feb 23;18:203. doi: 10.1186/s12864-017-3524-x (PMC5324198; doi:10.1186/s12864-017-3524-x)

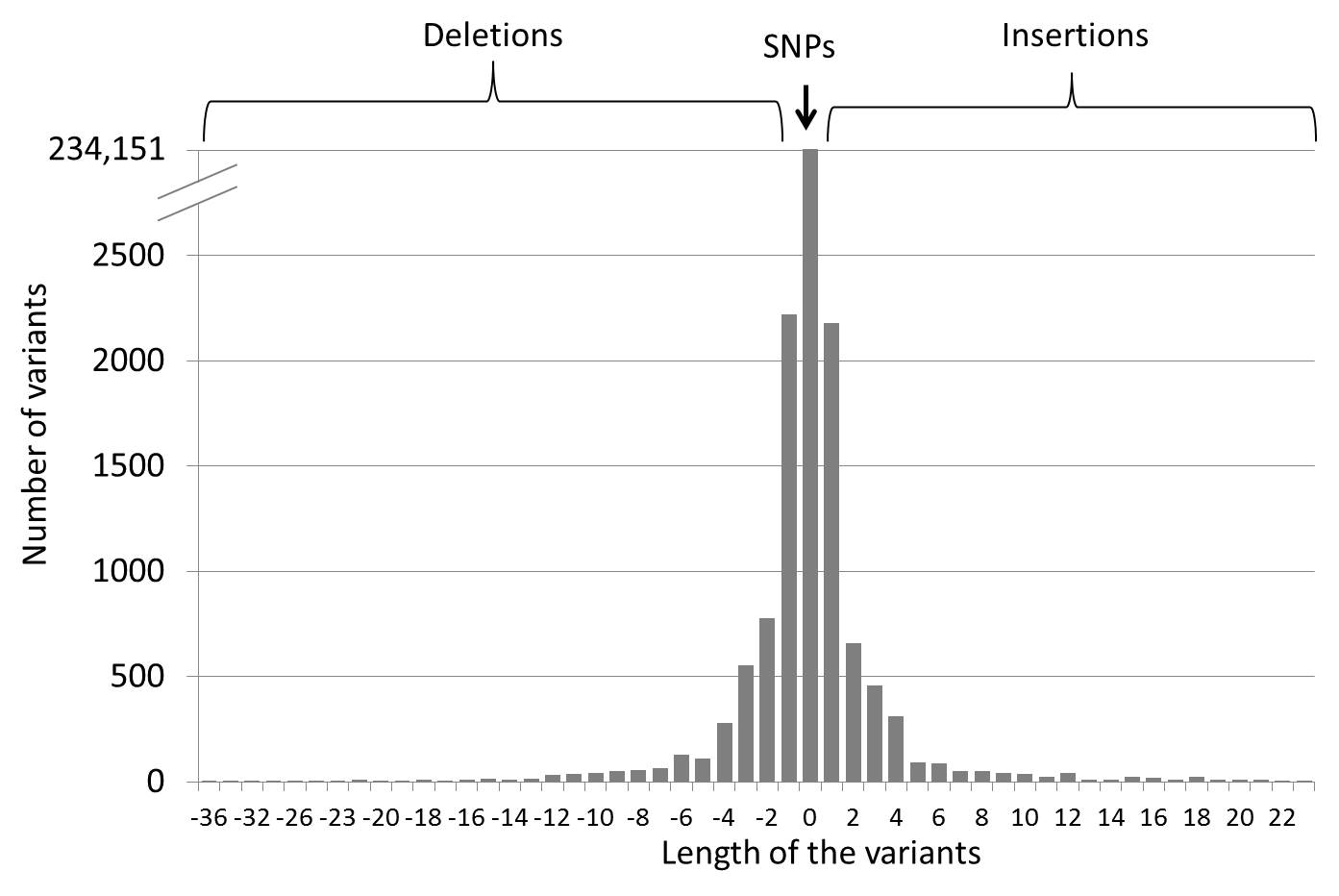

Supplement: Additional file 4: Figure S2. — Distribution of the length of the variants identified in this analysis. Polymorphisms of length equal to zero are SNPs. (JPG 65 kb) [file 12864_2017_3524_MOESM4_ESM.jpg]

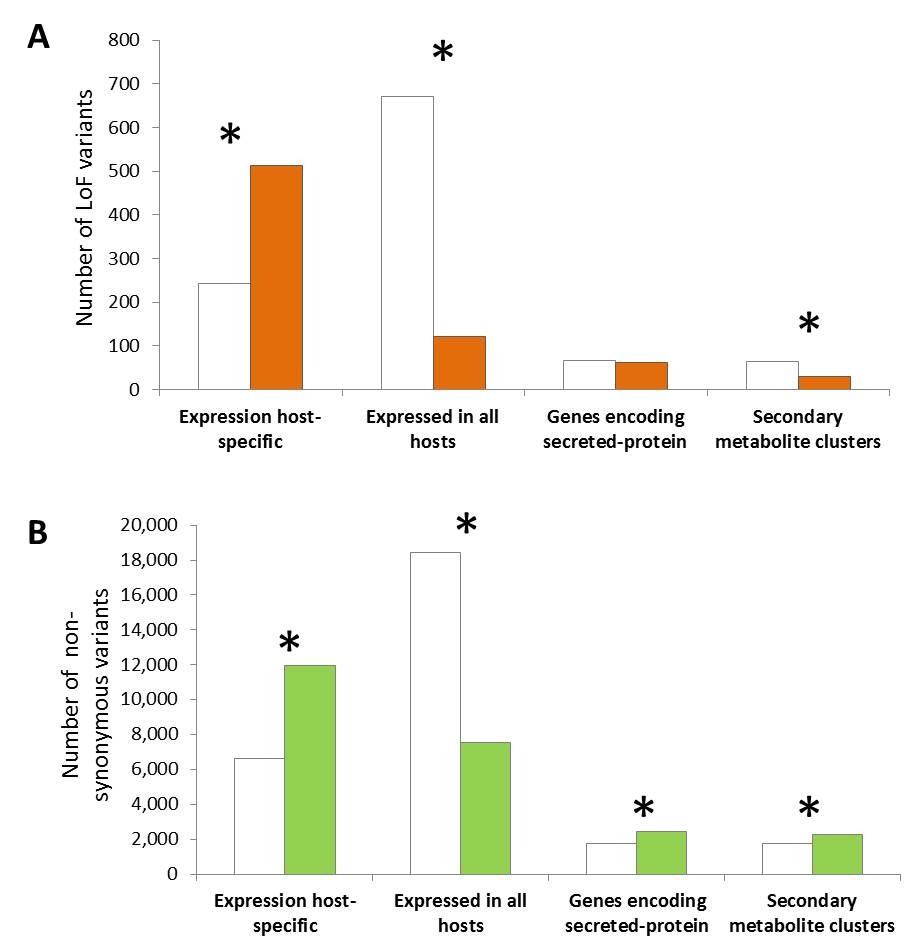

Supplement: Additional file 7: Figure S3. — Variant enrichment of genes with candidate functions. Representation of the observed variant number (in color) compared to the theoretical number expected under hypothesis of random distribution of variants (white). A. Variants predicted to lead to a loss of function of the protein. B. Variants predicted to have non-synonymous effects on the protein. “*” means Chi-squared test was significant (p-value < 0.001). (JPG 57 kb) [file 12864_2017_3524_MOESM7_ESM.jpg]
